# Supplementary material for: Protocol for the MicroRESUS study: The impact of circulatory shock and resuscitation on microcirculatory function and mitochondrial respiration after cardiovascular surgery
Source: PLoS One. 2022 Aug 26;17(8):e0273349. doi: 10.1371/journal.pone.0273349 (PMC9417024; doi:10.1371/journal.pone.0273349)
Supplement: S1 Appendix — (DOCX) [file pone.0273349.s001.docx]

# Supporting information (S1 Appendix)

**Power calculations and determination of sample size.** In the absence of information about what a normal range of sublingual microcirculatory parameters or mitochondrial respiration are in humans, our prospective cohort study was designed with the primary goal of building a reference range of these parameters for a cardiovascular patient population in a usual state of health (baseline measurements prior to surgery) as well as the relative change that occurs during a period that is high risk for circulatory shock. Observations of these changes will help us reach our primary goal of detecting changes in microcirculatory blood flow and mitochondrial respiration that contribute to the mechanism of shock.

We will enroll a total of 140 subjects in our prospective cohort study. This sample size reflects observations from preliminary data set of 20 subjects (unpublished) which found 15/20 subjects met our criteria for shock after cardiovascular surgery. We have chosen this sample size to reflect the largest number of patients needed to be enrolled to achieve adequate power for each study aim. Our enrollment target includes a 10% increase to account for potential loss to follow-up or incomplete data given the prospective cohort design. We have also built in a 5% allowance for exclusion due to inadequate microcirculation video quality.

**Power calculation 1: Assess the relationship of microcirculatory function with long-term clinical outcomes in patients undergoing cardiac surgery.** Thirty-day VVFDs will be compared using a Wilcoxon rank-sum test with a two-sided alpha of 0.05. Using preliminary data measuring the difference in preoperative sublingual perfused vessel density, we anticipate needing at least 134 subjects to detect a 2-day difference in 30-day VVFDs.

**Power calculation 2: Characterize changes in microcirculatory blood flow associated with Type A lactic acidosis after cardiac surgery.** In order to detect a significance with our multiple linear regression analysis including 6 predictors, we will need to enroll at least 98 subjects.

**Power calculation 3:** **Determine if patients with circulatory shock after cardiac surgery experience decreased oxygen utilization by measuring mitochondrial respiration using human blood cells.** In order to maintain power of 80% with a type-I error of 5%. We are expecting to see a 50% decrease in post-operative CI+CII OXPHOS in patients with shock. With an expected effect size of 1, our planned enrollment should detect a difference between both groups.
