# Supplementary material for: Protocol for the MicroRESUS study: The impact of circulatory shock and resuscitation on microcirculatory function and mitochondrial respiration after cardiovascular surgery
Source: PLoS One. 2022 Aug 26;17(8):e0273349. doi: 10.1371/journal.pone.0273349 (PMC9417024; doi:10.1371/journal.pone.0273349)
Supplement: Attachment — Submitted filename: PLOS One MicroRESUS Protocol Revisions.docx [file pone.0273349.s002.docx]

July 21, 2022

**Re: Protocol Manuscript submission to *PLOS ONE***

Dear *PLOS ONE* Editor(s):

Thank you for your thoughtful feedback on our protocol manuscript. We have attempted to address all of your concerns with careful modifications with additional explanations provided below. All changes within the manuscript are identifiable via track changes.

Overall, the most significant changes are:

* edited the document to meet the requested formatting requirements of the journal.

* we have addressed the questions about the study start time and

* adjusted the manuscript to specify cutoffs for normal and abnormal perfused vessel density and sample size calculations.

**Comments/Revision Requests**

1. Please ensure that your manuscript meets PLOS ONE's style requirements, including those for file naming.

   **RESPONSE:** The title page, author style, and manuscript style requests have been addressed.
2. Thank you for stating the following in the Acknowledgments Section of your manuscript: “Abramson & Emergency Medicine & Critical Care Research Fund”

   Please remove any funding-related text from the manuscript and let us know how you would like to update your Funding Statement.

   **RESPONSE:** The requested edits have been made to the manuscript document and cover letter. The revised Funding Statement reads: JCG is supported by the National Center for Advancing Translational Sciences of the National Institutes of Health, award number KL2TR001879. JCG is also supported by the University of Pennsylvania Abramson Emergency Medicine & Critical Care Research Fund. DHJ is supported by the National Heart, Lung, and Blood Institute of the National Institutes of Health, award number K08HL136858. The content is solely the responsibility of the authors and does not necessarily represent the official views of the National Institutes of Health.
3. We note that you have stated that you will provide repository information for your data at acceptance. Should your manuscript be accepted for publication, we will hold it until you provide the relevant accession numbers or DOIs necessary to access your data. If you wish to make changes to your Data Availability statement, please describe these changes in your cover letter and we will update your Data Availability statement to reflect the information you provide.

   **RESPONSE:** We have removed the Availability of data and materials from the declarations section since no preliminary data will be published with this manuscript. The appropriate DOI for future publications based on the results of this work is listed in the body of the manuscript under “Ethics and dissemination”.
4. One of the noted authors is a group or consortium “The MicroRESUS Investigator Group”. In addition to naming the author group, please list the individual authors and affiliations within this group in the acknowledgments section of your manuscript. Please also indicate clearly a lead author for this group along with a contact email address.

   **RESPONSE:** The consortium group has been removed, since only the listed authors were responsible for this publication.
5. Your ethics statement should only appear in the Methods section of your manuscript. If your ethics statement is written in any section besides the Methods, please delete it from any other section.
    **RESPONSE:** The ethics statement has been removed from ADDITIONAL INFORMATION and has been added to the body of the manuscript under “Ethics and dissemination” on *page 12****.***
6. Please include captions for your Supporting Information files at the end of your manuscript, and update any in-text citations to match accordingly.
    **RESPONSE:** The IRB approved document uploaded to “Supporting Information” has been moved to Other.
7. Additional Editor Comments: The authors are requested to make a number of changes in the manuscript. Of note, the study began several years ago. Can a reason be provided in the text for why the present manuscript was not developed and submitted sooner?

   **RESPONSE:** The concept for this study originated in 2020 and was submitted to our IRB for approval. We needed IRB approval to gather preliminary data (collected in late 2020) that was used to apply for the KL2 grant that is supporting the research project, which started in July 2021. The protocol manuscript was first written in the Summer/Fall of 2021 and underwent multiple internal revisions prior to submission to PLOS ONE in 2022**.**

Reviewer #1:

8. Thank you for this great study protocol article. I only wonder if measurements are also to be done during the operation since predominantly the start of CPB may be associated with microcirculatory derangement (Den Uil et al, J Thorac Cardiovasc Surg 2008). Good luck!

**RESPONSE:** We agree with the reviewer that initiation of cardiopulmonary bypass is strongly associated with microcirculatory injury and has been well described in the literature (den Os et al, Critical Care 2020). While adding this data point would likely extend findings of previous literature, we seek to concentrate on the primary research objective which focuses on the change during resuscitation after surgery. If and when resources are available to expand to intraoperative measurements, we will consider this addition. We have modified the study objective (Page 5) to ensure this is clear to readership.

Reviewer #2: The authors provided a study protocol for microcirculatory assessment in patients undergoing cardiac surgery. As the restoration of tissue perfusion is the primary goal of hemodynamic therapy, the study is of high interest. However, I have some questions and concerns about the manuscript and the study design:

9. The title of the study implies that you will investigate patients with shock. However, the inclusion criteria used do not include a marker for shock or hemodynamic instability. I think, only the minority of the included patients will suffer from shock. Please provide how shock will be defined in the study. What percentage of patients in your hospital develop shock during elective cardiac surgery procedures?

   **RESPONSE:** We appreciate this request for clarification. We have added our *a priori* definition of circulatory shock in the inclusion criteria. We have also included a revision of our sample size (page 6) and supporting information file that includes our power analyses to achieve our aims.
10. As primary endpoint you plan to use ventilator and vasopressor-free days. Further, you state the hypothesis that patients with poor post-operative microcirculatory function will have a higher degree of postoperative organ injury compared to patients with normal postoperative microcirculation. If you want to test this hypothesis, why do you not use a score for organ failure as the primary endpoint? Which parameters and cut-off values do you use to differentiate between normal and poor microcirculation?

    **RESPONSE:** Thanks for these important questions about injury endpoints. We have considered the choice of outcome carefully based on previously published data from our group as well as others.
    a. *Choice of primary endpoint:* We agree that organ failure scores are frequently higher after cardiovascular surgery, and that there are a number of already published manuscripts describing outcomes related to SOFA score (Pätilä et al, Annals of Thoracic Surgery 2006; Doerr et al, Journal of Cardiothoracic Surgery, 2011). We have also published on the changes in post-cardiac surgery SOFA and its association with changes in sublingual microcirculation (Greenwood et al, JCVA 2021). We have chosen to evaluate differences in VVFDs as the use of vasopressors and need for invasive/non-invasive mechanical ventilation are clinically important end-organ injuries that are frequent and also lead to prolonged ICU stay and have previously not been described. We have clarified this in the outcomes measures by specifying cardiovascular and pulmonary organ injury.
    b. *Poor vs. Normal Microcirculation cut-off:* Perfused vessel density is the imaging measurement of primary interest. We have added our cut-off values for MHI PVD (PVD ≤ 22 mm/mm^2^) and MHI (MHI > 0.4) based on our previously published work (Greenwood et al, Shock 2021). We have added these definitions to the **Outcome measures** on page 10.
11. In the statistics section you write "Continuous variables characterizing each study group will be reported as means with standard deviations or medians with interquartile ranges.". Which groups?

    **RESPONSE:** We have clarified data categories and added language to specify normal vs. non-normally distributed data on page 11.
12. You used a 2-day difference in ventilator and vasopressor-free days for sample size calculation. Which groups and what group sizes did you assume for this?

    **RESPONSE:** We will dichotomize patients into 2 groups based on perfused vessel density at ICU admission. We have clarified the group sizes based on preliminary data of PVD measurements on 20 post-operative patients.

Please let us know if you have any additional questions or concerns, we will be happy to address them.

Respectfully,
